# Book Review

**Published:** 1907-12

**Authors:** 


**Studies in the Bacteriology and Etiology of Oriental Plague.** By E. Klein, M.D., F.R.S. Pp. xv., 301. London: Macmillan and Co. 1906.—Those who were privileged to hear Dr. Klein's address to the Bristol Medico-Chirurgical Society on "Plague," in 1901, will gladly renew acquaintance with the subject in this volume, in which the whole subject of the etiology and intimate nature of plague is critically discussed. The bacteriology of the disease is first dealt with, including a description of the microbes which simulate in one or another respect the B. Pestis, and the methods and tests relied upon for discrimination. The important epidemiological relationships of plague in the rat to plague in man receive adequate notice; and it is to be noted that the author concludes that "there is a distinct failure of evidence that transmission of the disease is effected by fleas or lice from an infected animal to a healthy one." On this very important point Dr. Klein's opinion is not in accord with the results obtained in the remarkable and complete experiments recorded in the "Reports on Plague Investigations in India," issued by the Advisory Committee appointed by the Secretary of State for India, the Royal Society, and the Lister Institute, and printed in the September number of the *Journal of Hygiene*, 1906, which deserves the careful attention of all students of the subject.

**Tumours, Innocent and Malignant.** By J. Bland Sutton, F.R.C.S., Surgeon to and Member of the Cancer Investigation Committee of the Middlesex Hospital, &c. Fourth Edition. London: Cassell and Co. 1907.—This useful handbook still continues its successful career. Appealing alike to the surgeon and practitioner, it contains a large number of illustrations, which more or less convey the idea of the structures described. A considerable portion of the book is devoted to dental problems. The section on chorion-epithelioma has been brought up to date. It is evident that much of the text has been rewritten. Perhaps in the next edition it will be possible to harmonise the terminology of the surgeon with that of the academic pathologist.

**Opuscula selecta Neerlandicorum de arte Medica.** Pp. xii., 325. Amsterdam: F. van Rossen. 1907.—This volume, published by a committee to commemorate the Jubilee of the "Nederlandsch Tijdschrift voor Geneeskunde," contains reprints of four classical discourses, by Erasmus, by Boerhaave, by Gaubius, and the researches of Van Leeuwenhoek and Swammerdam. That of Desiderius Erasmus, dated Louvain, 13th March, 1518, is in praise of the art of Medicine. It is written in Latin, but an English translation faces each page. Leeuwenhoek's essay is in the form of a letter to the Royal Society of London, giving his views on the circulation of the blood, in 1688, clearly setting forth that the arteries and veins are continued blood-vessels. The other essays, written in Dutch, have French or German translations.

**Auscultation and Percussion.** By SAMUEL GEE, M.D. Fifth Edition. Pp. xviii., 325. London: Smith, Elder & Co. 1906.—The fifth edition of this classical work needs no further recommendation than to refer to the popularity which the previous editions have acquired. In the preface the author makes mention of skiagraphy in physical examination of the chest, and gives his reasons for not including the subject in this latest edition of his book, while at the same time he acknowledges the great value of the method. The alterations and additions to the text are very few, for indeed there was but little for Dr. Gee to modify or add to in the earlier editions of a work which will always remain to us as a model of literary style, combined with scientific accuracy and historical erudition. "Sunt clari hodieque et qui olim nominabuntur."

**First Lines in Midwifery.** By G. ERNEST HERMAN, M.B. Pp. xii., 222. New Edition. London: Cassell & Co., Limited. 1907.—The difficulty in a small book which deals with a large subject is to get the proper balance in what is necessarily an abstract. This is most successfully accomplished in this case, and, considering its small size, a remarkable résumé has been compiled. The illustrations are very numerous, and carefully chosen, and the text is written in a clear and simple style that is easy to follow. It would be well to recommend a doll as well as a skull for learning mechanisms with, especially to beginners. One wonders also why no mention is made of gentle massage of the breasts in helping to empty them for "caking" or "painful fulness," as it is such a useful adjunct. The book has been well known and appreciated for many years, and has already been reprinted and revised many times. The addition of a chapter on the requirements of the Central Midwives Board is a distinct advantage.

**Anæsthetics and their Administration.** By FREDERIC W. HEWITT, M.V.O., M.A., M.D. Third Edition. Pp. xxxiii., 627. London: Macmillan & Co., Limited. 1907.—The third edition of Dr. Hewitt's well-known work on *Anæsthetics and their Administration,* comes at a very opportune moment, seeing how much work has recently been done on the subject; and, as might be expected, this volume contains much new matter, dealing with such questions as ethyl chloride, surgical shock, acid intoxication, and the exact dosage of chloroform, and the author's views on these points are as welcome as they are fair and clear. We regret, however, to note the omission of an account of local and of spinal analgesia. In the chapter devoted to the "Physiology of Anæsthesia," special reference is made to the interesting researches of Hamilton Wright on the inimical effects of ether and chloroform upon the nerve cells of the brain and spinal cord; and a note has been made of the work of Kemp, Maunsell, and S. and H. Pringle, which shows that prolonged ether administration tends to seriously affect the organism. Under "Physiology of Chloroform Anæsthesia" has been added a concise description of the exact percentage apparatus of Waller, Dubois, and Collingwood; also a résumé of the researches by Embley and others, showing the depressing effect of lowered blood pressure upon the function of respiration; and the author points out that, according to Snow, primary cardiac paralysis from chloroform poisoning only occurs with high percentages of the drug, and that in the absence of such concentration the heart only fails secondarily to respiratory depression. The whole of Part II., dealing with the preparation of the patient, the selection of the anæsthetic, and the method and circumstances of administration has been very carefully rewritten, and much practical matter has been added. On page 375 the author discusses the Vernon Harcourt apparatus. On the whole, the volume combines the completeness of a reference book with the conciseness of a student's manual, and the fairness and impartiality of a standard work. And the preface should not be overlooked, as it is in itself an able appeal and an eloquent effusion.

**Anæsthetics, their Uses and Administration.** By DUDLEY WILMOT BUXTON, M.D., B.S. Fourth Edition. Pp. viii., 415. London: H. K. Lewis. 1907.—In re-editing his manual on anæsthetics, Dr. Dudley Buxton has succeeded in introducing much that is new without in any way interfering with the compactness and clearness which characterised the previous editions. Not only is the dosimetric method of administering chloroform described, but ethyl chloride is dealt with in full, and room has been found for a good account of local and spinal analgesia. As regards the fatalities under ethyl chloride, the author says that it is probable that this drug "is less safe than nitrous oxide, and must be placed between ether and chloroform in normal patients, but before ether when lung and kidney complications exist." On summing up the arguments in favour of spinal analgesia, Dr. Buxton says "those who have employed this method fail to show that it is safer than chloroform, or more free from unpleasant sequelæ," and he quotes Hare's opinion "that it is only applicable to cases for which general anæsthesia is an impossibility." Under local analgesia reference is made to the faintness and respiratory difficulty which are produced by excessive doses of adrenalin chloride, and for injection a solution of 1 in 200,000 is recommended. The present edition is very welcome, and will doubtless be even more popular than its predecessors.

**Guide to Anæsthetics.** By THOMAS D. LUKE, M.B. Third Edition. Pp. xvi., 136. Edinburgh: William Green & Sons.—This excellent little book has in four years reached its third edition, which fact in itself bears testimony as to its merits. As the present edition appears within two years of the previous one it has naturally been found unnecessary to make many changes in the text. We are of opinion that the section dealing with local anæsthesia should be more fully written up. There is every reason to think that Dr. Luke will soon be called on for the fourth edition.

**Golden Rules of Medical Evidence.** By STANLEY B. ATKINSON, M.A., M.B. Pp. 63. Bristol: John Wright & Co.—This little book is by one who combines in himself the qualifications of doctor and barrister. The subject lends itself well to summary in this way, and the book is really valuable. The hints are most excellent and practical, and should save many a mistake. It is just the thing to refer to when concerned in a medico-legal case, as it does not take ten minutes to look right through the book. A few of the aphorisms may be quoted as specimens: "Pregnancy must not be asserted until quickening has been felt, or the fœtal parts are palpable." "The body of the coroner's officer is always available for ocular demonstrations to the jury of the sites of injuries." "An early 'I don't know' is better than a late 'I did not know.'" "If you attend a court after being subpœnaed, the fee is due even should no evidence be called."

**Pulmonary Consumption.** By ARTHUR LATHAM, M.D. Pp. vii., 259. London: Baillière, Tindall & Cox. 1907.—A third edition of this work on the diagnosis and modern treatment of pulmonary consumption, with special reference to the early recognition and the permanent arrest of the disease calls for little comment. New sections have been inserted on such subjects as the value of the opsonic index in diagnosis and treatment, the use of Koch's new tuberculin in treatment, and Dr. Paterson's interesting observations on the value of manual labour at Frimley Sanatorium. The book is one which should be studied by all who are interested in questions relating to the sanatorium treatment of phthisis.

**Catalogue of the Pathological Museum of the Manchester University.** By J. LORRAIN SMITH, M.A., M.D. Manchester: Sherratt and Hughes, at the University Press.—To the Museum curator this catalogue must prove of good service. It is arranged upon the decimal system of classification, which provides the means of describing the anatomical and pathological appearances by means of decimals. In addition, however, the specimens are fully described. As this catalogue represents the contents of the

Manchester Pathological Museum, it must be conceded that the Northern University has an excellent collection of specimens illustrating morbid lesions. We thoroughly recommend the perusal of the catalogue to those who are arranging museums or undertaking original research. It is almost unnecessary to add that Lorrain Smith's part in the work represents the high standard of excellence which we are accustomed to expect from him.

**Aids to the Treatment of Diseases of Children.** By John McCaw, M.D., R.U.I., L.R.C.P. Edin. Third Edition. Pp. xiii., 383. London: Baillière, Tindall & Cox. 1907.—We are never quite sure whether we like least small books on big subjects, or big books on small subjects. The present volume may perhaps be included in the former class, as, although the title limits its scope to "Treatment," this cannot be considered to any advantage apart from diagnosis, symtomatology, pathology, &c.; in fact, on turning over the pages we find the ordinary arrangement is adopted, and each disease is considered under the above headings, treatment occupying no more than its usual share of the available space. Moreover, sufficient care has not been taken to exclude conditions which are dealt with in the ordinary text-books on medicine, and are not peculiar to or even common in childhood. Thus hydatid of the liver, cerebral tumour, Raynaud's disease, and amyloid disease are described in a manner too short to be of much value to the student. With the main part of the book, except for its brevity, we have little fault to find; there is a very succinct account of the principles of infant feeding and the digestive disturbances of young children, a section on the specific fevers, which does not differ much from that in most text-books of medicine, and a short account of circulatory and respiratory diseases. A propos of the latter, we are surprised to note that the author recommends aspiration in the treatment of empyema as a first resort. Other sections on blood, general and nervous diseases complete the book. There are, we suppose, some types of mind who are really "aided" by little books like this, and for them this one is sufficiently reliable. Judgment and experience, the essentials in the treatment of children's diseases, cannot be acquired from any book, but may certainly be materially assisted by some of the larger monographs on the subject. The book is well got up and printed, and has an adequate index.

**St. Bartholomew's Hospital Reports.** Vol. XLII London: Smith, Elder & Co. 1907.—There are many valuable and interesting papers in this volume of the reports. We may refer to a few which have specially interested us, but there are many others of equal importance, and amongst these we may mention a very exhaustive one by Dr. Finlay Alexander on "Hypertrophic Pulmonary Osteo-Arthropathy," with several good skiagraphic plates. Altogether readers of this volume, whether their interests are medical or surgical, will find much to interest them. Dr. Herbert Williamson records two cases of large, partly solid and partly cystic, embryomata, one occurring in a girl of 16, and the other in a young man of 23, which on microscopic examination showed various elements of complex tissue, such as bone, cartilage, nervous tissue, and tissue resembling intestinal mucous membrane, as well as tissue quite like that of ordinary sarcoma and carcinoma. He refers to other cases of these "embryomata" published during recent years, and describes their character and symptoms, and relation to other forms of growth. His communication is one of considerable value. Mr. Elmslie contributes a paper on "Late Rickets: or the Continuation of Early Rickets to an unusually late age." He regards the changes in the epiphyses at the wrist as the essential condition in rickets, and refuses to recognise a case of genu valgum, even with marked bony deformity, occurring over ten years of age, as late rickets at all. We fail to see why we should thus limit the term "late rickets" to cases with epiphyseal enlargement only. Of what nature, we should be inclined to ask, does he consider the disease which manifests itself as marked deformity in the bones of the limbs in young persons from ten to twenty years of age? He gives us in his paper a table of cases of late rickets accompanied by epiphyseal enlargement. Mr. Faulder, who has been studying bronchoscopy at the clinic of Prof. Killian, of Freiburg, writes an interesting account of its use and the indications for it. The great difficulty he admits is to get the needful experience. He says, "Evidently the best way is to practise on a living subject, if such be found." We hardly think such could or should be found for the manipulative practise of the method which involves passing the instrument through the larynx into the tracheæ. Its value in practised hands seems to be great in detecting foreign bodies in the bronchus, and he tells us of a case in which a stricture of the bronchus was seen and dilated with its aid.

**Diseases of Women.** By GEORGE ERNEST HERMAN, M.B. Third Edition. Pp xvi., 900. London: Cassell & Company Limited. 1907.—The present edition of this well-known work has been thoroughly revised and brought up to date. The plan of the book is substantially the same as in previous editions. Chapters II.—V. are devoted to a consideration of the principal symptoms of which gynæcological patients complain; and although this arrangement has its drawbacks, it is one which should prove very useful to students and practitioners. With regard to the treatment of uterine displacements, the author considers ventrofixation the most satisfactory operation for prolapse, and that of vaginal fixation for retroflexion. For intractable cases of chronic salpingo-oophoritis, removal of the uterus and appendages through the vagina is recommended; we think, however, that in most cases the abdominal operation would be the easier and safer.

The chapters on malignant disease of the uterus have been enlarged, and a fuller description of its pathology added. There is also a good account of chorio-epithelioma. For cases of fibromyoma of the uterus requiring operation the author recommends abdominal hysterectomy; myomectomy he considers has a very limited field of usefulness, and oophorectomy is obsolete. In Chapter I. a good account of the pathology of vascular caruncle is given. Throughout the book special attention is devoted to the clinical aspects of diseases of women, and the details of treatment and operative measures are fully described. The illustrations throughout are numerous and well executed, and the letterpress is excellent.

**Annual Report of the Board of Regents of the Smithsonian Institution for the year ending June 30th, 1905.** Washington: Government Printing Office. 1906.—This is not such a bulky volume as usual, but the papers which it contains are of the same absorbing interest that we are accustomed to. The papers dealing with medical subjects are a paper read before the Indian section of the Society of Arts in May, 1905, by Dr. Creighton on "Plague in India," in which he points out that plague especially affects villages where the inhabitants live in dwellings the walls of which are made with mud, and that there can be no real cure for the devastations of the disease without a more civilised kind of dwelling; and one on the "Fight against Yellow Fever," by A. Dastre, showing the success that has been obtained in stamping out yellow fever by waging war against the special mosquito which is the cause of the disease. Another medical paper is "Progress in Radiography," by L. Gastine, written in 1905. Since this date still further progress has been made in this science, and it is interesting, on looking over a paper written only two years since, to see how X-ray methods continue to be improved. A very curious paper to read at the present day is a "History of Photography," which consists of extracts from a *Manual of Photography* by Robert Hunt, published in 1854, and is devoted to giving an account of Sir John Herschell's researches into the effect of light on substances other than silver salts—such as the cyanides—and his efforts to get a workable photographic process from the same. Papers on the "Genesis of the Diamond," "Gold in Science and in Industry," and "Liberia" may be picked out as well worth reading; but perhaps the paper of all others which appealed to us was the paper read before the Royal Geographical Society in February, 1905, by Sir Frank Younghusband on the "Geographical Results of the Tibet Mission." The enthusiastic way he describes the scenery passed through, the way the members of the expedition bore their privations, and the successful accomplishment of the expedition, makes one of the most refreshing bits of reading that we have come across for a long time.

